# Sonic hedgehog through Gli2 and Gli3 is required for the proper development of placental labyrinth

**DOI:** 10.1038/cddis.2015.28

**Published:** 2015-02-19

**Authors:** Y B Pan, Y Gong, H F Ruan, L Y Pan, X K Wu, C Tang, C J Wang, H B Zhu, Z M Zhang, L F Tang, C C Zou, H B Wang, X M Wu

**Affiliations:** 1 Department of Pharmacology, School of Medicine, Zhejiang University, Hangzhou, China; 2 Department of Gynaecology and Obstetrics, The First Affiliated Hospital, School of Medicine, Zhejiang University, Hangzhou, China; 3 Department of Internal Medicine, The Affiliated Children Hospital, School of Medicine, Zhejiang University, Hangzhou, China; 4 State Key Laboratory of Reproductive Biology, Institute of Zoology, Chinese Academy of Sciences, Beijing, China

## Abstract

Sonic hedgehog (Shh) functions as a conserved morphogen in the development of various organs in metazoans ranging from *Drosophila* to humans. Here, we have investigated the potential roles and underlying mechanisms of Shh signaling in murine placentation. Immunostaining revealed the abundant expression of the main components of Shh pathway in both the trophectoderm of blastocysts and developing placentas. Disruption of Shh led to impaired vascularogenesis of yolk sac, less branching and malformation of placental labyrinth, thereby leading to a robust decrease in capacity of transplacental passages. Moreover, placenta-specific gene incorporation by lentiviral transduction of mouse blastocysts and blastocyst transplantation robustly knocked down the expression of Gli3 and Gli2 in placenta but not in embryos. Finally, Gli3 knockdown in *Shh*^−/−^ placentas partially rescued the defects of both yolk sac and placental labyrinth, and robustly restored the capacity of transplacental passages. Gli2 knockdown in *Shh*^+/^^−^ placentas affected neither the capacity of tranplacental passages nor the vascularogenesis of yolk sac, however, it partially phenocopied the labyrinthine defects of *Shh*^−/−^ placentas. Taken together, these results uncover that both Shh/Gli2 and Shh/Gli3 signals are required for proper development of murine placentas and are possibly essential for pregnant maintenance.

Placenta is a transient and the first organ of embryonic origin, connecting the fetus to the maternal body to allow nutrient uptake and waste elimination.^1^ The development of functional mouse placenta begins from blastocyst at E3.5 when the formation of the trophectoderm layer,^2^ and then the labyrinth begins to form at E8.5 when the allantois attaches to the chorion, followed by the branching of the chorion, fetal vascularogenesis, and formation of a maternal blood circulation.^3, 4^ By around E14.5, the mature placenta comprises three layers, trophoblast gaint cell (TGC) layer, spongiotrophoblast (SP) layer, and labyrinthine (Lb) layer. The TGC layer is composed of multiple subtypes of trophoblast giant cells, whereas the SP layer is composed of SPs and glycogen trophoblast cells. The mouse Lb layer is composed of an intricate network of maternal and fetal blood vessels (FBVs) separated by a placental barrier that consists of four cellular layers: sinusoidal TGCs (STGCs) line the maternal lacunae, layer I and II syncytiotrophoblasts (ST-I and ST-II) form syncytial layers, and endothelial cells (ET) surround the fetal vessels.^3, 5^

Hedgehog (Hh) family of secreted proteins including sonic hedeghog (Shh), indian hedgehog (Ihh), and desert hedgehog (Dhh) have conserved roles in development of various organs and cells from *Drosophila* to humans.^6^ In vertebrates, Hh binding to the receptor patched (Ptc) relieves the repression of co-receptor smoothened (Smo), leading to the activation of downstream Gli transcription factors including Gli1, Gli2, and Gli3. Gli1 functions exclusively as a transcriptional activator, whereas Gli2 and Gli3 contain both activator and repressor forms.^7, 8^ In the absence of Hh, Gli3 is proteolytically cleaved into C-terminally truncated repressor form (Gli3R), and Gli2 is targeted for degradation. In the presence of Hh, the ensuing Hh signaling blocks the cleavage of Gli3 into Gli3R and stabilizes Gli2 that can then be N-terminally truncated into its activator form (Gli2A).^9^ Overall, the conserved effect of Hh is to switch the Glis from repressors into activators and allow for well-coordinated transcriptional events.^7^

Cholesterol modification of Hh ligands is fundamental for Hh activity and its modulation by Ptc; cholesterol biosynthesis is also required for intracellular Hh signaling transduction.^10, 11, 12^ *Nsdhl* gene encodes a sterol dehydrogenase involved in cholesterol biosynthesis, and deficiency of *Nsdhl* gene severely impairs Ihh and Ptc expression. Defects of Hh signaling in the *Nsdhl* knockout mice are coupled with malformation of murine placenta, providing the first clue that Hh signaling is critical for the development of placenta.^13^ However, Hh signals controlling the placental development still lacks the direct evidence. Moreover, owing to their inability to undergo vascular remodeling, *Ihh*^–/–^ yolk sacs form fewer and smaller blood vessels, whereas *Smo*^–/–^ yolk sacs arrest at an earlier stage of vascularogenesis, and fail to undergo even the limited vascular remodeling observed in the *Ihh*^–/–^ yolk sacs,^14, 15^ prompting us to hypothesize that in addition of Ihh, other Hh ligands could contribute to the proper development of yolk sac as well as placenta. Hence, we investigated the expression patterns of the main components of Hh pathway, and further dissected the roles and underlying mechanisms of Shh signaling in developing placentas by using *Shh* knockout mice and placenta-specific gene incorporation.

## Results

### Localization of the main components of Shh pathway in mouse placenta

**Figure 1:**
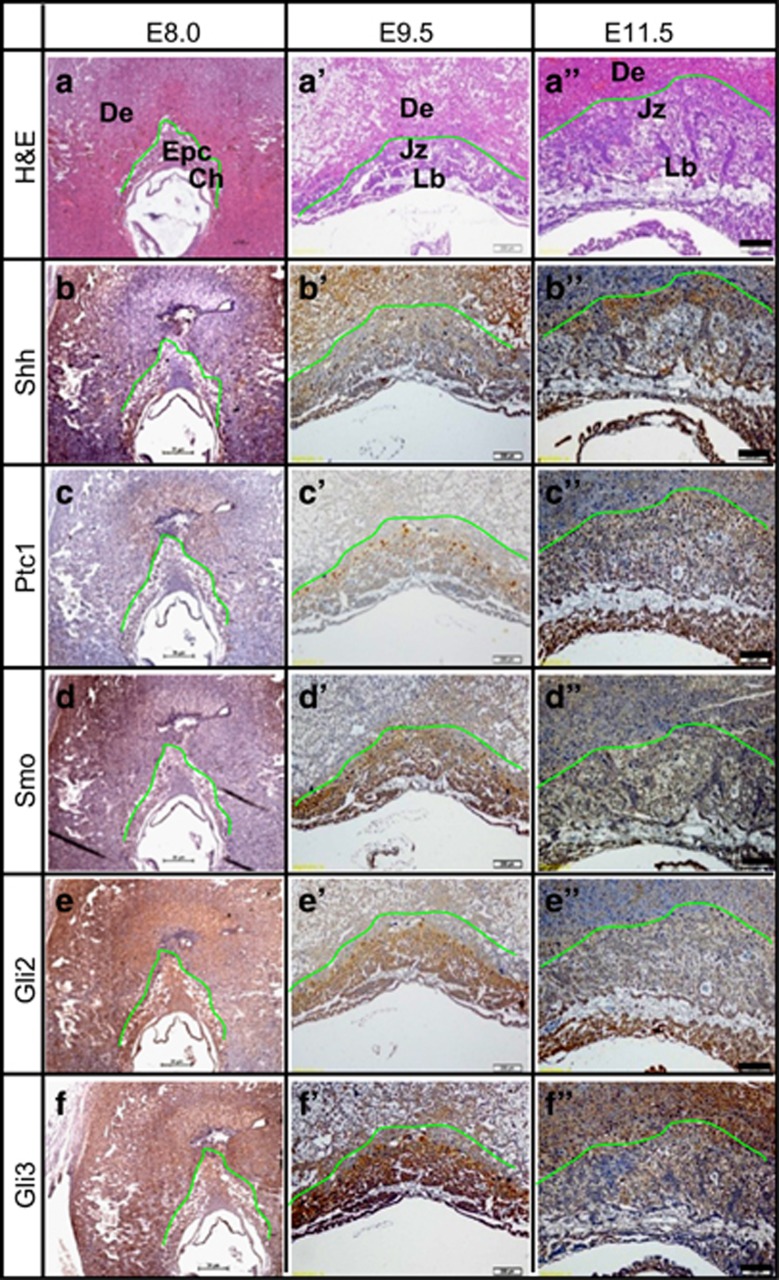
The localization of main components of Shh pathway in developing placenta. (**a-a**'') Top panels show the H&E staining of serial paraffin-embedded sections of E8.0–E11.5 placentas, and were designated the areas presented on the corresponding panels below. (**b-f**'') Immunohistochemistry staining for the main components of Shh pathway, including Shh, Ptc1, Smo, Gli2, and Gli3, was performed in the serial sections. De, decidium; Ch, chorion; Epc, ectoplacental cone; Jz, junctional zone (TGC layer and SP layer); Lb, labyrinth. Scale bar: 200 μm

To examine the localization of the main components of Shh pathway, we performed immunostaining for the blastocysts at E3.5 and placentas from E8.0 to E11.5. In blastocysts, Shh and Ptc1 were relatively restricted in the trophectoderms, caudal-related homeobox 2 (Cdx2), a trophoblast stem cell marker, was evenly expressed in both the trophectoderms and inner cell masses (ICMs), while octamer-binding transcription factor 3/4 (OCT3/4), an embryonic stem cell marker, was more highly expressed in ICMs than in the trophectoderms (Supplementary Figures 1a–d”). Shh-derived immunohistochemistry signal was detectable in the primary TGC and chorion at E8.0, and was diffusely localized in decidium, junctional (TGC and SP layers) and labyrinthine zones at E9.5, whereas it was relatively restricted in the placenta but not in the decidium at E11.5 (Figures 1a–b''). Ptc1 and Smo were barely detectable at E8.0, whereas they were abundantly and region-specifically localized at placentas of E9.5 and E11.5; Ptc1 was more highly expressed in junctional zone than in labyrinthine zone, whereas Smo was almost evenly expressed in both of them (Figures 1c–d”). Gli2 was broadly but not cell lineage specifically localized in the uterus and placenta at E8.0, and was diffusely distributed in all three layers of murine placentas at E9.5 and E11.5 (Figures 1e–e”). Gli3 was diffusely expressed in uterus and placenta, especially in ectoplacental cone (EPC) at E8.0, however, it was robustly localized in both the junctional zone and labyrinthine zone at E9.5 and E11.5 (Figures 1f–f”). To gain quantitative information about the expression levels, we performed RT-PCR assays in developing placentas from E8.5 to E11.5. Dhh and Ihh were expressed at relatively higher levels than Shh at E8.5 and E9.5, but Shh was expressed at a higher level than either Dhh or Ihh at E11.5; Ptc1, Gli2, and Gli3 were expressed at relatively higher levels than Smo (Supplementary Figures 1e and f). Overall, the main components of Hh pathway are abundantly expressed in murine developing placentas.

### Placenta-specific knockdown of Gli3 and Gli2

**Figure 2:**
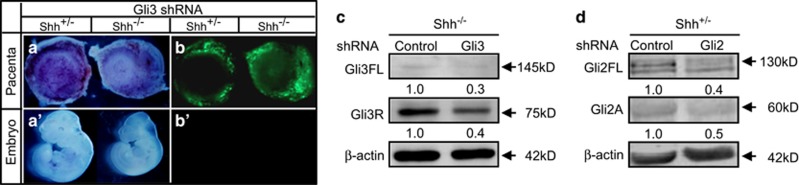
Placenta-specific knockdown of Gli3 and Gli2 by lentiviral transduction. (**a**–**b**') Gross appearance of placenta and embryo under light and fluorescent stereoscopy. (**c** and **d**) Western blot assays for the full-length Gli3 (Gli3FL), Gli3 repressor (Gli3R), full-length Gli2 (Gli2FL) and Gli2 activator (Gli2A) in placentas (E12.0) infected with either scribble(control)-shRNA- or Gli3/Gli2-shRNA-expressing lentiviruses. The mean intensity of the first band was set to 1.0

Gli3 predominantly acts as a repressor in Hh pathway, genetic inactivation of Gli3 is able to rescue Shh mutant phenotypes in neural tube, limb, face, forebrain, and skin. Conversely, Gli2 predominantly functions as an activator in Hh pathway, Gli2 knockdown is able to inactivate Hh signaling and mimic the phenotypes of Shh mutant.^16, 17, 18, 19^ To determine whether Gli3 and Gli2 mediate Shh-controlling placentation, we performed placenta-specific gene incorporation by lentiviral transduction of blastocysts (after removal of zona pellucid) and blastocyst transplantation to knock down the Gli3 and Gli2 expression in *Shh*^−/−^ and *Shh*^+/^^−^ placentas, respectively. Green fluorescent protein (GFP)-carrying and shRNA-expressing lentiviruses were successfully introduced into trophectoderm but not ICM of blastocyst, and led to robust GFP expression in all of placentas analyzed but none of the fetuses (Gli3-shRNA expressing lentivirus as a representative, Figures 2a–b'). The Gli3-shRNA decreased the protein levels of full length of Gli3 (Gli3FL) by 70% and Gli3R by 60% in *Shh*^−/−^ placentas as compared with the scribble(Control)-shRNA (Figure 2c), whereas the Gli2-shRNA led to decreases in the protein levels of full length of Gli2 (Gli2FL) by 60% and Gli2A by 50% in *Shh*^+/^^−^ placentas (Figure 4d).

### Expression patterns of the main components of Shh pathway in placentas after genetic modifications

**Figure 3:**
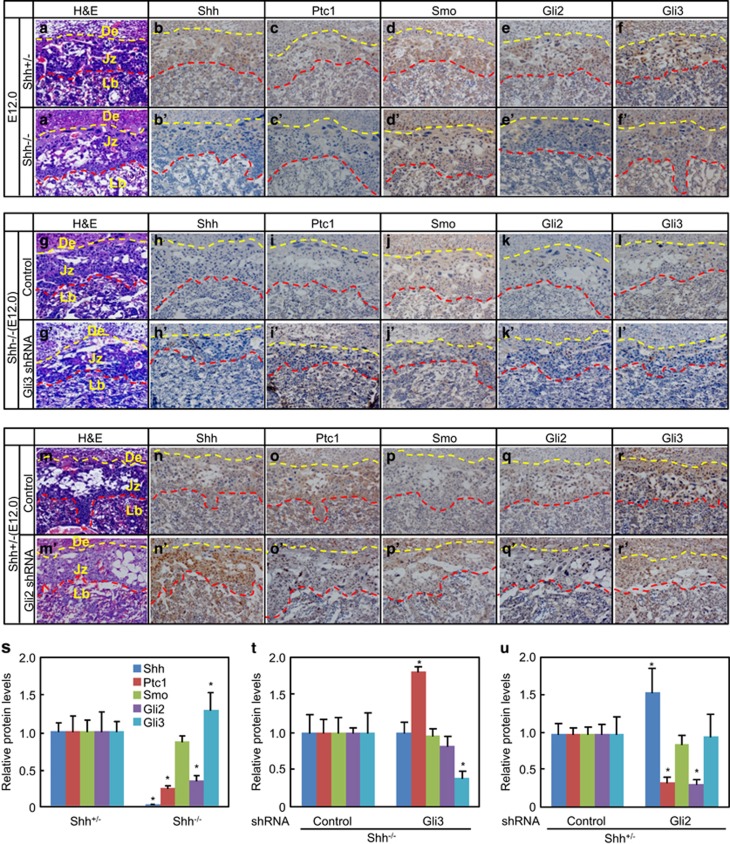
Expression patterns of the main components of Shh pathway in placentas (E12.0) after genetic modifications. (**a**–**f**' and **s**) *Shh*^*+/−*^ and *Shh*^−/−^ placentas, (**g**–**l'**, and **t**) *Shh*^*−/−*^ placentas infected with either control-shRNA- or Gli3-shRNA-expressing lentiviruses, and (**m**–**r**' and **u**) *Shh*^*+/−*^ placentas infected with either control-shRNA- or Gli3-shRNA-expressing lentiviruses. Placentas were subjected to serial paraffin-embedded sections and were stained for the main components of Shh pathway by immunohistochemistry. The quantitative histomorphometry was performed by using the Osteomeasure Analysis System, the immunohistochemistry signals from respective control placentas were defined as 1. *P<0.05 *versus* respective controls

To investigate the expression patterns of the main components of Shh pathway, we harvested the *Shh*^*+/−*^ or *Shh*^−/−^ placentas infected with or without either scribble-shRNA- or Gli2/Gli3-shRNA-expressing lentiviruses at E12.0 and performed the immunohistochemistry staining. In *Shh*^−/−^ placentas as compared with in *Shh*^*+/−*^ ones, Shh and Ptc1 were barely detectable, Gli2 was robustly decreased in junctional zone and labyrinth, Gli3 appeared a slight increase in labyrinth, while Smo exhibited no obvious difference (Figures 3a–f' and s). In *Shh*^−/−^ placentas, Gli3-shRNA-expressing lentiviruses led to significant increases in Ptc1 of junctional zone and labyrinth and expected decreases in Gli3 of whole placentas as compared with scribble-shRNA-expressing lentiviruses (Figures 3g–I' and t). In *Shh*^*+/−*^ placentas, Gli2-shRNA-expressing lentiviruses resulted in significant decreases in Ptc1, expected decreases in Gli2, and unexpected increases in Shh of junctional zone as compared with scribble-shRNA-expressing lentiviruses (Figures 3m–r' and u). Thus, either *Shh* knockout or Gli2 knockdown in placentas effectively attenuates the Hh signaling, whereas knockdown of Gli3 partially restores the Hh signaling in *Shh*^−/−^ placentas.

### Morphological and functional changes in placenta and yolk sac after genetic modifications

**Figure 4:**
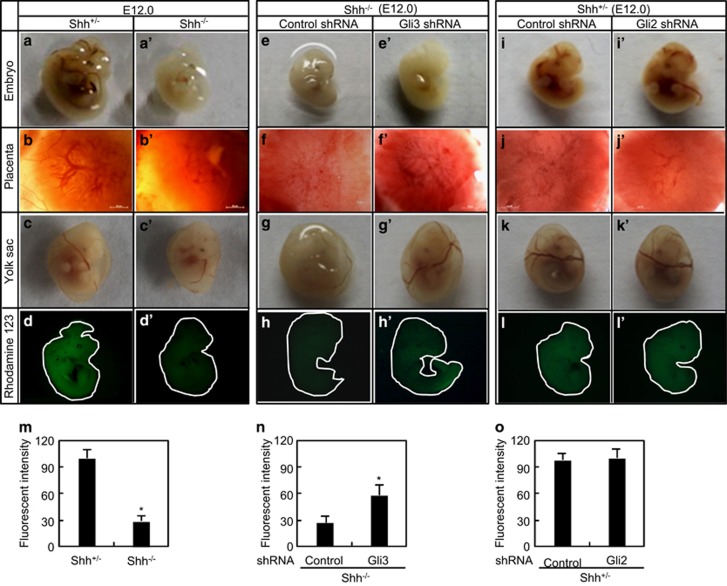
Morphological and functional defects in the embryos and placentas after genetic modifications. (**a**–**c**', **e**–**g**' and **i**–**k**') Gross appearance of embryos, placentas, yolk sacs at E12.0. (**d**–**d**', **h**–**h**', **l**–**l**' and **m**–**o**) The transplacental passage of Rhodamine 123. The pregnant dams were intraperitoneally injected with rhodamine 123 at 1 mg/kg, after 2 h, the living embryos at E12.0 were visualized under a fluorescence stereomicroscope and the fluorescent intensities were determined by spectrophotofluorometer (*n*=3). *P<0.05 *versus* respective controls

To investigate the morphological alterations after various genetic modifications, we harvested the embryos, placentas, and yolk sacs at different embryonic stages and measured transplacental passage by injecting the pregnant dams with the fluorescent dye. Loss of Shh caused fetal growth retardation and embryonic lethality at around E13.5 as described previously.^20, 21^ At E12.0, the *Shh*^−/−^ embryos exhibited smaller and paler than *Shh*^+/^^−^ ones, and the blood vessel branching at the surface of the yolk sac and on the fetal side of the placenta was barely visible in Shh null alleles (Figures 4a–c'), suggesting that loss of Shh leads to severe defects in vascularization of both embryos and extraembryonic annexes. Measurement of transplacental passage revealed a significant decrease in the fluorescence recovered in *Shh*^−/−^ embryos as compared with in *Shh*^+/^^−^ ones (Figures 4d, d' and m). As expected, Gli3 knockdown in placentas did not obviously rescue the abnormality of *Shh*^−/−^ embryos, but markedly ameliorated the defects in the blood vessels branching at the surface of yolk sacs and on the fetal side of placentas in *Shh*^−/−^ mutants (Figures 4e–g'). Measurement of transplacental passage revealed that Gli3 knockdown in *Shh*^−/−^ placentas led to a significant increase in the fluorescence recovered in *Shh*^−/−^ embryos (Figures 4h, h' and n). Finally, Gli2 knockdown in *Shh*^+/^^−^ placentas led to neither the obvious abnormality of embryos nor the defects in the blood vessels branching at the surface of the yolk sacs, whereas it markedly reduced the vascularization on the fetal side of *Shh*^+/^^−^ placenta (Figures 4i–k'). Unexpectedly, measurement of transplacental passage revealed that Gli2 knockdown in *Shh*^+/^^−^ placentas did not cause an obvious change in the fluorescence recovered in *Shh*^+/^^−^ embryos (Figures 4l, l' and o).

### Histological analysis of placentas after various genetic modifications

**Figure 5:**
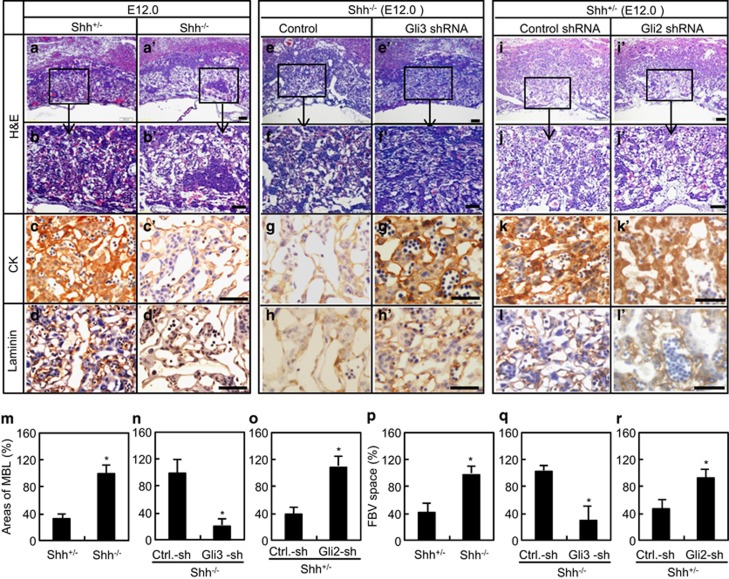
Histological analysis of placentas after genetic modifications. (**a**−**b**', **e**−**f**' and **i**−**j**') H&E staining of placentas after genetic modifications. (**c**−**d**', **g**−**h**' and **k**−**l**') Immunohistochemistry staining for cytokeratin (CK) and laminin in placentas. CK marks maternal blood lacunae (MBL) and laminin defines fetal blood vessel (FBV) spaces. (**m**−**r**) Relative areas of MBL and FBV spaces. Scale bar: 50 *μ*m. The relative areas arisen from *Shh*^*−/−*^ placentas were defined as 100%, *P<0.05 *versus* respective controls

To examine the phenotypes of placentas after various genetic modifications, we performed histological analyses. At E12.0, the *Shh*^−/−^ placentas appeared no gross abnormality, but a little bit thicker and bigger than *Shh*^+/^^−^ ones. Though the junctional and labyrinthine zones were well organized in the *Shh*^−/−^ placentas (Figures 5a and a'), striking phenotypes occurred in labyrinth, exhibiting loose architecture with enlarged porous aspects, dilated maternal blood lacunae (MBL), enlarged FBV spaces, and sparse trophoblastic layers that separated the MBL from FBV (Figures 5b, b'). Immunostaining further revealed a dilated MBL surrounded by the epithelial-derived chorionic-trophoblast cells, cytokeratin (CK)-positive cells, and a reduction in the number of FBV lined by allantoic mesenchymal cells, laminin-positive cells (Figures 5c–d'), suggesting that severe defects in the branching of labyrinth existed in the *Shh*^−/−^ placentas, and possibly contributed to the reduction of transplacental transport. However, 5-bromodeoxyuridine (BrdU) incorporation assay and caspase-3 staining indicated that these defects were not associated with the proliferation and apoptosis (Supplementary Figures 2a–d').

There was no gross difference between the *Shh*^−/−^ placentas infected with scribble-shRNA and Gli3-ShRNA, however, placentas infected with Gli3-shRNA became more compact than those infected with scribble-shRNA (Figures 5e and e'). The labyrinth in *Shh*^−/−^ placentas with Gli3 knockdown exhibited close texture with less porous aspects, smaller sizes of MBL and FBV space separated by well-defined trophoblastic layers (Figures 5f, f'). Immunostaining further revealed almost normally shaped and sized MBL surrounded by normal number of CK-positive cells, and well-defined FBV space surrounded by normal number of laminin-positive cells (Figures 5g–h'), suggesting that severe defects in the branching of labyrinth in *Shh*^−/−^ placentas were almost completely rescued by Gli3 knockdown.

No gross difference existed in *Shh*^+/^^−^ placentas infected with either scribble- or Gli2-ShRNA (Figures 5i and i'). However, *Shh*^*+/*^^−^ placentas with Gli2 knockdown had the similar architecture to *Shh*^−/−^ placentas, exhibiting the enlarged MBL and FBV space, disarrangement of trophoblastic cell layers (Figures 5j, j'). Immunostaining further revealed a dilatation in the MBL lined by CK-positive cells and a reduction in the number of the FBV and laminin-positive cells (Figures 5k–l'), suggesting that *Shh*^+/^^−^ placentas infected with Gli2-shRN possessed similar defects in the branching of labyrinth to those of *Shh*^−/−^ placentas.

### Expression of trophoblast lineage differential markers in placentas after genetic modifications

**Figure 6:**
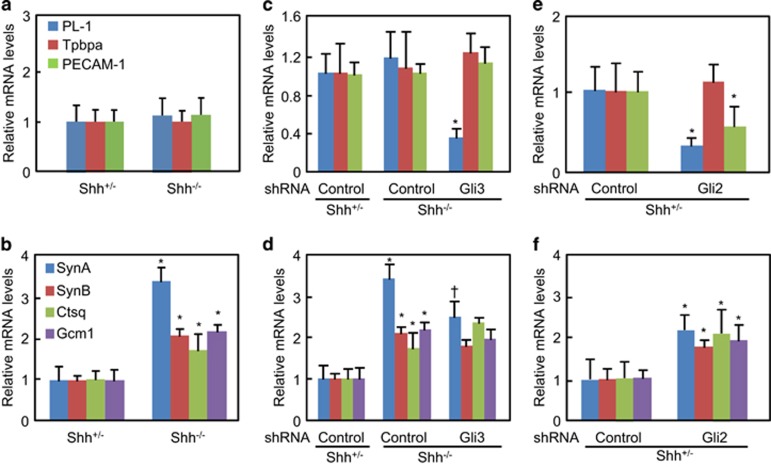
Quantitative RT-PCR assays for the mRNA levels of trophoblast lineage differential markers of placentas (E12.0) after genetic modifications. (**a**–**e**) The mRNA levels of PL-1, Tpbpa, and PECAM-1 were determined in the placentas after various genetic modifications. (**b**–**f**) The mRNA levels of SynA, SynB, Ctsq, and Gcm1 were determined in the placentas after various genetic modifications. PL-1, Tpbpa, PECAM-1, SynA, SynB, Ctsq, and Gcm1 were the respective markers for trophoblast gaint cells, spongiotrophoblasts, endothelial cells, layer I syncytiotrophoblasts, layer II syncytiotrophoblasts, sinusoidal trophoblast gaint cells, and syncytiotrophoblasts. The relative mRNA levels arisen from respective controls were defined as 1, *P<0.05; ^†^*P*<0.05 *versus* respective controls

To further analyze the placental defects, we performed quantitative RT-PCR to determine the mRNA levels of differential markers. Neither prolactin-like protein 1 (PL-1) and trophoblast-specific protein alpha (Tpbpa), the respective markers of TGC and SP, nor the platelet EC adhesion molecule (PECAM)-1, an endothelial marker, exhibited significant difference between *Shh*^*+/*^^−^ and *Shh*^−/−^ placentas (Figure 6a). Unexpectedly, in *Shh*^−/−^ mutants, cathepsin Q (Ctsq), syncytin A (SynA), syncytin B (SynB), the respective markers for STGC, layer I and layer II syncytiotrophoblasts, and glial cells missing 1 (Gcm1), a transcriptional factor of syncytiotrophoblasts, were significantly upregulated (Figure 6b). Moreover, Gli3 knockdown in *Shh*^−/−^ placentas robustly decreased the mRNA levels of PL-1 but not Tpbpa and PECAM-1, and significantly attenuated the Shh null-producing increases in mRNA levels of SynA but not SynB, Ctsq, and Gcm-1 (Figures 6c and d). Finally, Gli2 knockdown in *Shh*^+/-^ placentas robustly decreased the mRNA levels of PL-1 and PECAM-1 but not Tpbpa, and significantly increased them of SynA, SynB, Ctsq, and Gcm-1 (Figures 6e and f), suggesting that Gli2 could be essential for TGC differentiation and labyrinthine morphogenesis, and that Gli2 knockdown in *Shh*^+/^^−^ placentas partially phenocopies the defects of *Shh*^−/−^ placentas.

### Fine structure of placental labyrinth after genetic modifications

**Figure 7:**
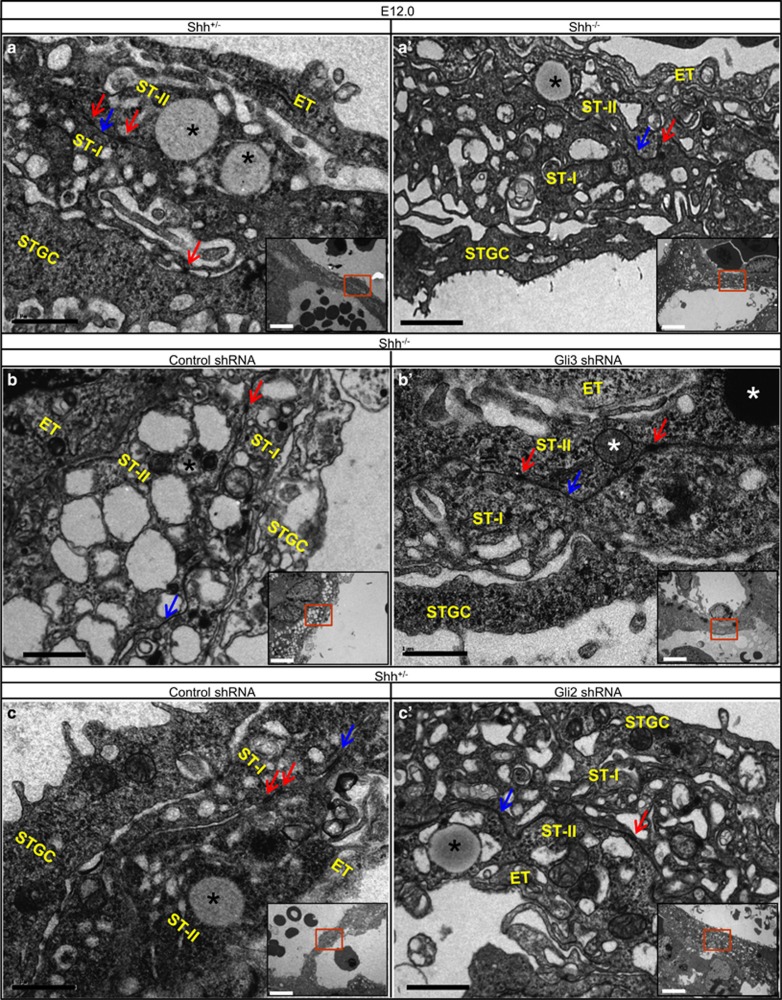
Transmission electron microscopic results of maternofetal interface in labyrinth of placentas after genetic modifications. (**a**and**a**') *Shh*^*+/−*^and *Shh*^*−/−*^ placentas, (**b**and**b**') *Shh*^*−/−*^ placentas infected with either control-shRNA- or Gli3-shRNA-expressing lentiviruses, (**c**and**c**') Shh^*+/−*^ placentas infected with either control-shRNA- or Gli2-shRNA-expressing lentiviruses. Lipid inclusions (asterisk); Gap junctions (arrow); Desmosomes (arrowhead)

To further dissect the placental defects, we examined the fine structure of the interface between MBL and FBV by using transmission electron microscopy. The labyrinth from *Shh*^+/−^ placenta showed the structure of typical trilaminar interhemal barrier (TIB) consisting of an STGC layer and ST-I and ST-II syncytiotrophoblast layers that lined the fetal endothelium and separated the MBL from the fetal capillary (Figures 7a and c). The two layers of STs tightly adhered to each other through frequent desmosomes and gap junctions (Figures 7a and c). The labyrinth from *Shh*^−/−^ placentas showed the shrunken fetal endothelium with a continuous basement membrane surrounding ST-II, shrunken STGC layer lacking of cell protrusions, disarranged ST-I layer sparsely attached to STGC layer and filled with many enlarged cytoplasmic vacuoles that were considered to reduce the intracellular transport,^5, 22, 23^ and disarranged ST-II layer with sparse lipid inclusions (Figure 7a'). In some cases, the TIB of *Shh*^−/−^ placentas exhibited normally shaped fetal endothelium surrounding ST-II, normal shaped STGC layer lacking of cell protrusions, thin ST-I layer filled with excessive cytoplasmic vacuoles and thickened ST-II layer with excessive cytoplasmic vacuoles and sparse lipid inclusions (Figure 7b). Moreover, in *Shh*^−/−^ mutants, the trophoblast layers had sometimes lost many of their frequent desmosomes and gap junctions that were responsible for intercellular transport between ST-I and ST-II (Figures 7a' and b). In *Shh*^−/−^ placentas, Gli3 knockdown was able to rescue the abnormally shaped fetal endothelium, markedly restored the ill-defined TIB, exhibiting normally sized and shaped STGC layer with re-emergence of cell protrusions, fully recovered the number of cytoplasmic vacuoles of ST-I and ST-II layers and the numbers of lipid inclusions of ST-II, and ameliorated frequent desmosomes and gap junctions between ST-I and ST-II (Figure 7b'). In *Shh*^+/^^−^ placentas, Gli2 knockdown led to ill-defined TIB that was similar to that in *Shh*^−/−^ placentas, exhibiting shrunken STGC layer with exiguous cell protrusions, net-shaped ST-I layer filled with large vacuoles, relatively normal ST-II layer with less lipid inclusions (Figure 7c'). The interface of STGC and ST-I layer in Gli2 knockdown placentas was not clearly visible, and the frequent desmosomes and gap junctions between ST-I and ST-II existed but were fewer (Figure 7c'). Thus, Shh knockout in the placentas leads to the disturbance of TIB, and Gli3 knockdown in *Shh*^−/−^ placentas almost completely rescues the abnormality of TIB, while Gli2 knockdown in *Shh*^*+/−*^ placentas results in the morphological defects similar to those in *Shh*^−/−^ placentas in some aspects.

## Discussion

By genetic mouse models and placenta-specifically lentiviral incorporation, to our best knowledge, the present study for the first time reveals the important roles of Shh singling in the morphogenesis of murine placenta. Shh signaling through Gli2 and Gli3 controls the proper development of placental labyrinth and maintains the placental barrier for fetomaternal exchanges.

The abundance of the main components of Hh pathway in both the trophectoderm of blastocysts and developing placentas implicates that by autocrine and paracrine mechanisms, Hh signals regulate placental development. Region-specific localization of Ptc1, Smo, Gli2, and Gli3 prompts us to speculate that placental trophoblasts are the Hh-responding cells, and Shh acts on these Hh-responding cells to activate the signaling pathway and promote the differentiation of them. Though previous studies have demonstrated that Wnt/β-catenin signaling is essential for ensuring blastocyst competency to implantation,^24^ and that Hh signaling functions as a upstream of Wnt/*β*-catenin signaling,^25, 26^ Shh null mutants seemed to form morphologically normal blastocysts and implant normally, suggesting that Shh signaling might not contribute to the implantation of blastocysts. Though Shh, Dhh, and Ihh have overlapping functions in the morphogenesis,^6, 7^ knockout of Shh alone is sufficient to induce the defects of placentation, suggesting that Shh, Dhh, and Ihh could have distinct roles. Additionally, though Dhh and Ihh are expressed more than Shh in the middle stage (E8.0 and E9.5) of gestation, from E11.5 the Shh is expressed more than either Ihh or Dhh. Since we mainly focus on the placental development in the late stage, we have chosen the Shh as the target in the present study.

Previous studies have shown that *Smo*^–/–^ yolk sacs failed to undergo even the limited vascular remodeling observed in the *Ihh*^–/–^ yolk sacs,^14,15^ whereas in the present study, we have demonstrated that Shh null yolk sacs have a severe defect in vascularogenesis. Thus, Shh and Ihh could have an overlapping role in vascularogenesis of yolk sacs, and the complementary effects of Shh might make the phenotypes of vascularogenesis in *Smo*^–/–^ yolk sacs more severe than in *Ihh*^–/–^ ones. Inactivation of Shh signaling is associated with the defects in vascular development,^27,28^ and activation of Shh signaling causes hypervascularization,^29^ however, the precise mechanism governing the vascularogenesis by Shh signaling is still not clear.^30,31^ In current study, Shh null led to severe defects in vasculature of yolk sacs and branching of FBV but not in the PECAM-1 expression, and knockdown of Gli2 but not Gli3 expression has obvious effects on the PECAM-1 expression, suggesting that Shh/Gli3 but not Shh/Gli2 functions as the player for the vascular remolding (branching) rather than for the formation of blood vessels.

Most importantly, by lentiviral incorporation into blastocysts and blastocyst transplantation, we have succeeded in knocking down the expression of Gli3 and Gli2 in placentas. In vertebrates, Shh signaling is mediated by Gli1, Gli2, and Gli3, and the functions of three Gli proteins overlap but also are distinct.^32,33^ Both Gli1 and Gli2 are transcriptional activators, whereas Gli3 functions as a transcriptional repressor.^34,35^ The current study suggests that Gli3 is a likely downstream target of Shh signaling in controlling the placental morphogenesis. Beyond the placenta as reported here, Shh/Gli3 signaling has been implicated in the patterning and development of other tissues, such as limb bud, telencephalic-diencephalic junction, olig2+ neurons, and lung.^36, 37, 38, 39^ However, Gli3 knockdown in *Shh*^−/−^ placentas led to decreases in mRNA levels of PL-1 that was not affected by mere Shh knockout, suggesting that Gli3 acts as a downstream target of not only Shh signal but also Ihh and Dhh signals. On the other hand, the current study suggests that Gli2 is also a likely downstream target of Shh signal in placentation. Beyond the placenta as reported here, Shh/Gli2 signaling has also been implicated in patterning and development of other tissues such as mouse embryos, skeleton, spinal cord, and hair follicle.^40, 41, 42, 43^ However, Gli2 knockdown in *Shh*^+/-^ placentas led to significant decreases in mRNA levels of PL-1 and PECAM-1 that were not affected by mere Shh knockout, suggesting that Gli2 also acts as not only Shh signal but also Ihh and Dhh signals. Finally, Gli2 knockdown in *Shh*^+/−^ placentas did not cause the phenotypes as same as in *Shh*^−/−^ ones. One possible explanation for this discrepancy is that Gli2 may act as a downstream of Ihh and Dhh except for Shh, alternatively, giving both Gli2 and Gli1 function as activators in Hh signaling, the overlapping roles of them could be another explanation.

In the present study, the observed defects in transport across the maternal-fetal interface of placentas with different genotypes are significant findings. We suggest that defects in both the blood vessel branching and TIB in *Shh*^−/−^ placentas contribute to the disruption of transplacental transport and fetal growth retardation, and that Shh signal is essential for maintaining the function of labyrinthine two distinct cell lineages (trophoblast and mesoderm) that differentiate to trophoblasts and fetal capillary endothelium, respectively.^44,45^ Giving Gli3 knockdown has been able to markedly rescue the defects in transplacental transport of *Shh*^−/−^ placentas, we suggest that Gli3 functions as a downstream of Shh signal and has essential roles in blood vessel branching and proper construction of TIB. Though Gli2 knockdown in *Shh*^*+/*^^−^ placentas leads to severe morphological defects of TIB, it affects neither the vascularization of yolk sac nor the transplacental transport. A possible explanation for the discrepancy is that morphological alterations of TIB in placentas with Gli2 knockdown are not sufficient to induce the defect in tranplacental transport. Further experiments are required to address this issue.

Overall, by genetic mouse models and placenta-specifically letiviral incorporation, the present study suggests that Shh/Gli2 and Shh/Gli3 signaling are essential for proper development of murine placentas and are possibly important for pregnant maintenance.

## Materials and Methods

### Mouse strains

*Shh*^+/^^–^ mice on a C57BL/6J genetic background were generated as previously described,^20, 21^ and were obtained from Cyagen Biosciences (Santa Clara, CA, USA). Adult wild-type and *Shh*^+/–^ mice were housed at Zhejiang University Animal Care Facility according to the institutional guidelines for laboratory animals. The animal protocol was approved by the Zhejiang University Institutional Animal Care and Use Committee. Founder mice and their progenies were genotyped by PCR using tail genomic DNA. Females were mated with fertile males of heterozygous mice to induce pregnancy; the day that the vaginal plug was first observed was considered day 1 of pregnancy. Pre-implantation embryos at blastocyst stages were harvested by flushing the uterus as appropriate, and the littermates were harvested from E8.0 to E12.0 for analyses of phenotypes of placenta and embryo as well.

### Hematoxylin and eosin staining, immunohistochemistry, and BrdU labeling

Fresh placentas were fixed in 10% neutral buffered formalin (NBF) at 4°C and embedded in paraffin, and serial sections (4 μm) were stained with hematoxylin & eosin (H&E). Immunohistochemistry staining was performed by using the SP Rabbit HRP Kit (Kangwei Reagents, Beijing, China) according to the manufacturer's instructions. Briefly, placental sections were deparaffinized and rehydrated in xylene and a graded series of ethanol, and then were subjected to antigen retrieval in 10 mM sodium citrate and 10 mM citric acid. Tissue sections were incubated with 3% H_2_O_2_ in methanol for 15 min to quench endogenous peroxidase followed by sequential incubation with normal serum for 30 min, control IgG and primary antibodies overnight at 4°C, and HRP-labeled secondary antibody for 30 min. The diaminobenzidine (DAB) solution was used for development of brown color, and the sections were counterstained with hematoxylin. The primary antibodies were as follows: anti-Shh (1:100, NG1796045, Millipore, Billerica, MA, USA), anti-Ptc1(1:100, ab53715, Abcam, Cambridge, UK), anti-Smo (1:100, ab72130, Abcam), anti-Gli2 (1:100, ab26056, Abcam), anti-Gli3(1:100, ab69838, Abcam), anti-Cytokeratin (1:100, Z0622, Dako, Glostrup, Denmark), anti-Laminin (1:100, L9393, Sigma-Aldrich, St. Louis, MO, USA), anti-Caspase3 (1:100, aBS1518, Bioworld Technology, St. Louis Park, MN, USA). BrdU incorporation analysis was performed by intraperitoneal injection of BrdU (100 mg/kg of body weight) into pregnant dams, 2 h before killing. Placentas were harvested, fixed, embedded, sectioned, and subjected to immunostaining by using BrdU Immunohistochemistry Kit (Millipore) according to the manufacturer's instructions. The quantitative histomorphometry was performed using the Osteomeasure Analysis System (OsteoMetrics, Inc., Decatur, GA, USA) as described previously.^46^

### Generation of Gli2-shRNA- and Gli3-shRNA-expressing lentiviruses and placenta-specific knockdown of Gli2 and Gli3

The Gli2-shRNA- and Gli3-shRNA-expressing lentiviruses were generated as described previously.^47^ Briefly, for construction of lentiviral shRNA-expressing vectors, the hairpin shRNA templates of complementary oligonucleotide containing overhangs were digested. The synthesized complementary oligonucleotides were annealed and inserted into a lentiviral shRNA-expressing vector, pSicor-GFP. The sense and anti-sense sequences for mGli2 and mGli3 were listed in Supplementary Table S1. 293FT packaging cells were transfected with 6 *μ*g of each construct by Lipofectamine reagents (Life Technologies, Grand Island, NY, USA), 72 h after transfection, lentivirus-containing supernatants were harvested, and the lentiviruses with titers more than 1 × 10^7^ CFU/ml were used for infection. Placenta-specific knockdown of Gli2 and Gli3 was performed as described previously.^48,49^ Briefly, blastocysts generated from *Shh*^+^^/–^ intercrosses were harvested on E3.5, and zona pellucidae were removed with acidic Tyrode's solution (Sigma, St. Louis, MO, USA). Each blastocyst was incubated for 8 h in a 6 μl M16 medium droplet (3 x 10^6^ transduction units/ml). Transduced blastocysts were observed under a laser scanning confocal microscopy or implanted into pseudopregnant females. Eight blastocysts were transplanted into each horn of the uterus. Mice were killed at different stages of pregnancy, and the whole placenta and fetus were visualized under a fluorescence stereomicroscope. The placentas were subjected to various analyses, and the embryos were used for genotyping.

### Whole mount immunofluorescence

Immunofluorescence staining in blastocysts was performed as described previously.^50^ Briefly, blastocysts were fixed in 10% NBF at room temperature for 10 min, permeabilized in 2.5% Tween-20 in PBS for 5 minutes, and then incubated overnight at 4°C with primary antibodies against Shh (1:50), Ptc1 (1:50), Cdx2 (1:50, 932-392M-EN, XBioGenex), or Oct3/4 (1:50, sc-5729, Santa Cruz Biotechnology). After washes with PBS, blastocysts were incubated with Alexa Fluor 546-conjugated second antibody (Invitrogen, Grand Island, NY, USA) for 1 h at room temperature. As controls for specificity, some blastocysts were stained with the secondary antibody only, and the controls were always negative. The nuclear counterstaining was performed by using 4',6-diamidino-2-phenylindole (DAPI), and the fluorescence signals were visualized under a confocal laser microscope (Zeiss LSM510, Oberkochen, Germany).

### Transplacental passage of Rhodamine 123

Transplacental passage of Rhodamine 123 was performed as described previously.^5^ Briefly, pregnant dams from heterozygous mating were intraperitoneally injected with Rhodamine 123 (Dojindo Laboratories, Kumamoto, Japan) at 1 mg/kg of body weight. Mice were killed 2 h after injection, and the living embryos were visualized under a fluorescence stereomicroscope (SMZ1500, Nikon, Tokyo, Japan). After that, the embryos were homogenized in the lysis buffer and subjected to determination of fluorescent intensity by spectrophotofluorometer (excitation 485 nm, emission 520 nm), the fluorescent intensity was normalized to the protein content.

### Quantitative RT-PCR and western blot

Total RNA was isolated from placenta by using TRIzol reagent (Invitrogen), and 1 *μ*g each of total RNA was used to synthesize cDNA. Messenger RNA levels of trophoblast markers including PL-1, Tpbpa, Ctsq, syncytin A, syncytin B, and endothelium marker P-ECAM1/CD31 were determined by quantitative RT–PCR as described previously.^51^ The relative amounts of the mRNA levels of various markers were normalized to the GAPDH levels, respectively, and the relative difference in mRNA levels was calculated by 2^–ΔΔCt^ method. The primers for quantitative real-time PCR were listed in Table S2. Western blot was performed as described previously.^51^ Briefly, the placental lysates were prepared by using radioimmunoprecipitation assay lysis buffer, after homogenization and centrifugation, and the lysates containing 40 *μ*g of protein were subjected to SDS-PAGE and transferred onto a PVDF membrane. Immunoblotting was performed with anti-Gli2 (1:1000) and anti-Gli3 (1:1000) primary antibodies, and immunofluorescent second antibody, and the signals were visualized with Odyssey Infrared Imaging System (LI-COR, Lincoln, NE, USA). *β*-Actin (Santa Cruz) was used as internal standards. Image software (ImageJ, http://rsb.info.nih.gov/ij/download.html) from National Institutes of Health was used to quantify the immunoreactive bands, and the mean intensity of the first band was set to 1.

### Transmission electron microscopy

Placentas were fixed with 2.5% glutaraldehyde in 0.1 M cacodylate buffer for 36 h at 4 °C. Specimens were post-fixed with 2% osmium tetroxide in 0.1 M cacodylate buffer for 2 h and stained with 2% uranyl acetate in 0.1 M cacodylate buffer-30% methanol for 1 h. Samples were then dehydrated through a graded series of 30–100% ethanol and 100% propylene oxide and embedded in Epon812 (Electron Microscopy Sciences, Hatfield, PA, USA). Ultrathin sections (80 nm) were contrasted with 4% uranyl acetate and 0.25% lead citrate and then examined with an FEI Technai 12 microscope (FEI Corporate, Hillsboro, OR, USA) operated at 80 kV. Semi-quantification for the size and number of cells, the numbers of vacuoles and lipid inclusions, and the numbers of the gap junctions and desmosomes was performed in the images, which were acquired digitally from a randomly selected pool of 10–15 fields under each condition. Experiments were performed at the Facility Core of Microscopic Imaging, Zhejiang University School of Medicine.

### Statistical analyses

All the numerous data are expressed as the mean±S.E.M., and were analyzed by *t* test or one-way ANOVA and Student's *t* test (SPSS 13.0J software; SPSS, Inc., Chicago, IL, USA). Statistical significance was assessed at *P*<0.05. Experiments were independently triplicated, and results were qualitatively identical. Representative experiments are shown.

## Abbreviations

Hh: hedgehog
Shh: sonic hedgehog
Ihh: indian hedgehog
Dhh: desert hedgehog
TGC: trophoblast gaint cell
SP: spongiotrophoblast
Lb: labyrinth
STGC: sinusoidal trophoblast gaint cells
ST-I: layer I syncytiotrophoblasts
ST-II: layer II syncytiotrophoblasts
ET: endothelial cells
Ptc1: patched-1
Smo: smoothened
Gli3FL: full-length of Gli3
Gli3R: Gli3 repressor form
Gli2FL: full-length of Gli2
Gli2A: Gli2 activator form
Cdx2: caudal-related homeobox 2
OCT3/4: octamer-binding transcription factor 3/4
EPC: ectoplacental cone
BrdU: 5-bromodeoxyuridine
PL-1: prolactin-like protein 1
Tpbpa: trophoblast-specific protein alpha
PECAM-1: platelet endothelial cell adhesion molecule
Ctsq: cathepsin Q
SynA: syncytin A
SynB: syncytin B
Gcm1: glial cells missing-1
TIB: trilaminar interhemal barrier
