# Supplementary material for: Sonic hedgehog through Gli2 and Gli3 is required for the proper development of placental labyrinth
Source: Cell Death Dis. 2015 Feb 19;6(2):e1653–. doi: 10.1038/cddis.2015.28 (PMC4669788; doi:10.1038/cddis.2015.28)
Supplement: Supplementary Figures [file cddis201528x1.ppt]

## Slide 1
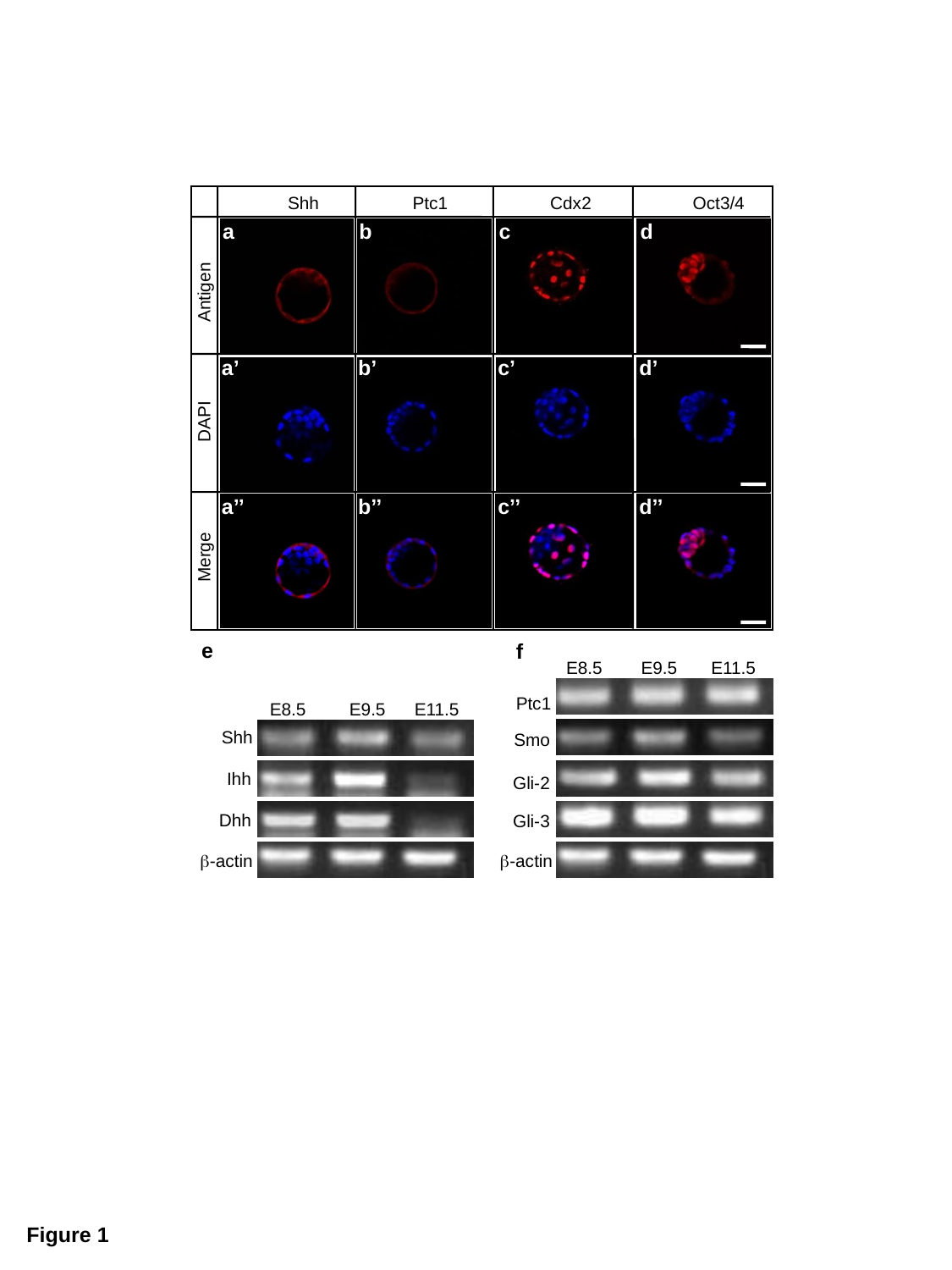

Shh
 Ptc1
Cdx2
Oct3/4
a
b
c
d
Antigen
a’
b’
c’
d’
DAPI
a’’
b’’
c’’
d’’
Merge
e
f
E8.5 E9.5 E11.5
Ptc1
E8.5 E9.5 E11.5
Shh
Smo
Ihh
Gli-2
Dhh
Gli-3
β-actin
β-actin
Figure 1

## Slide 2
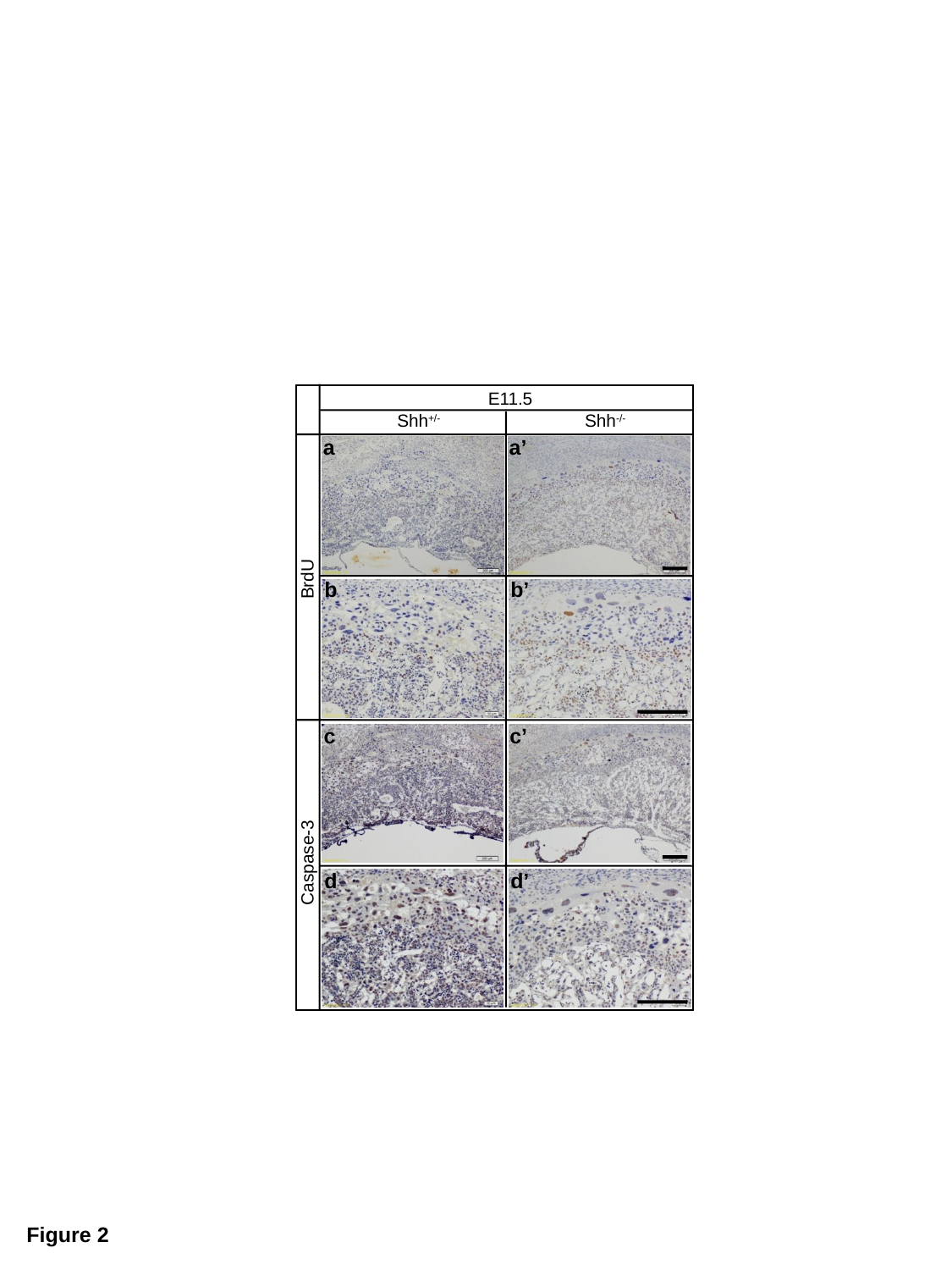

E11.5
Shh+/-
Shh-/-
a
a’
BrdU
b
b’
c
c’
Caspase-3
d
d’
Figure 2
