# Supplementary material for: Sonic hedgehog through Gli2 and Gli3 is required for the proper development of placental labyrinth
Source: Cell Death Dis. 2015 Feb 19;6(2):e1653–. doi: 10.1038/cddis.2015.28 (PMC4669788; doi:10.1038/cddis.2015.28)
Supplement: Supplementary Tables [file cddis201528x2.ppt]

## Slide 1
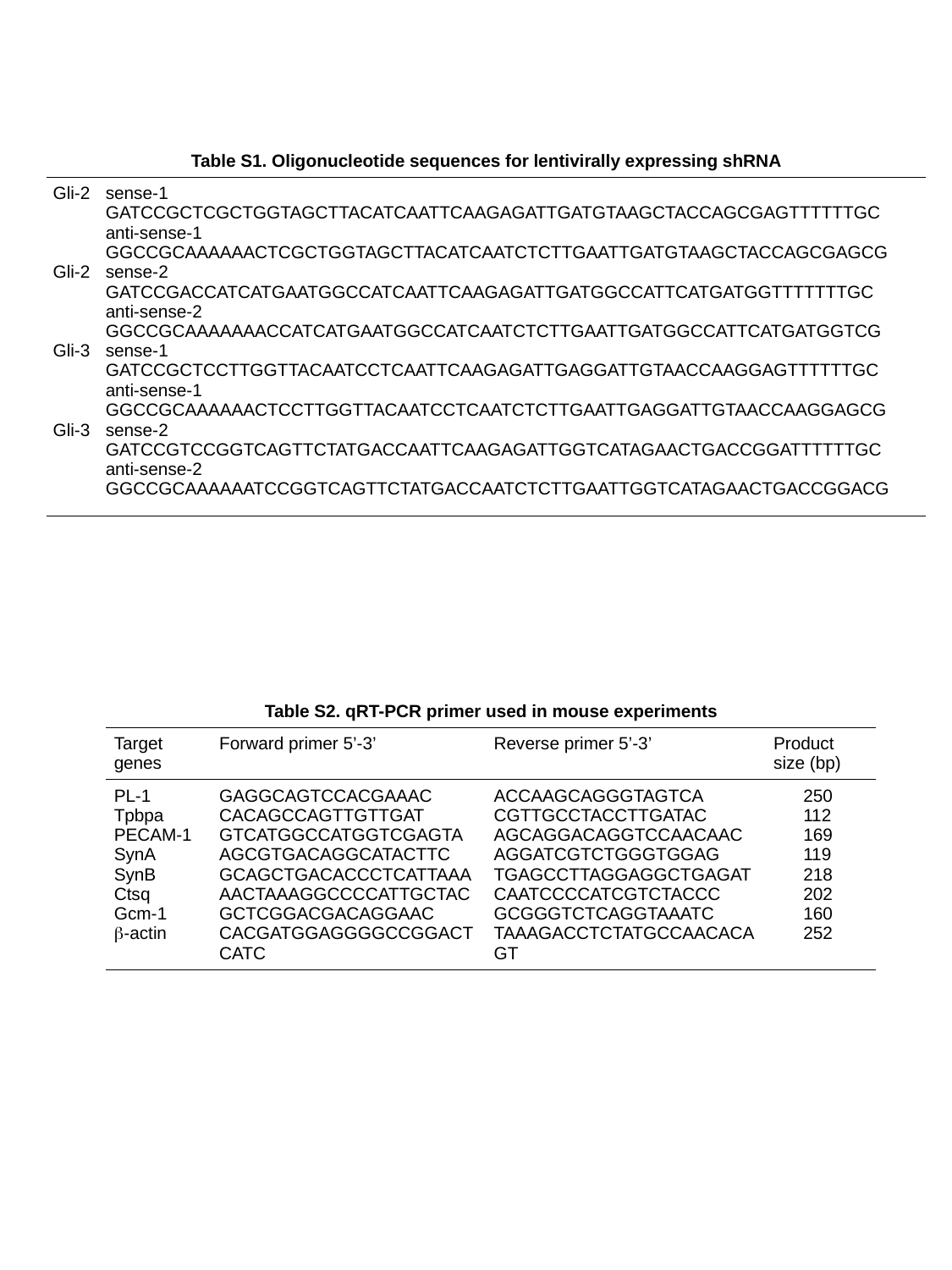

| Table S1. Oligonucleotide sequences for lentivirally expressing shRNA | |
| --- | --- |
| Gli-2 Gli-2 Gli-3 Gli-3 | sense-1 GATCCGCTCGCTGGTAGCTTACATCAATTCAAGAGATTGATGTAAGCTACCAGCGAGTTTTTTGC anti-sense-1 GGCCGCAAAAAACTCGCTGGTAGCTTACATCAATCTCTTGAATTGATGTAAGCTACCAGCGAGCG sense-2 GATCCGACCATCATGAATGGCCATCAATTCAAGAGATTGATGGCCATTCATGATGGTTTTTTTGC anti-sense-2 GGCCGCAAAAAAACCATCATGAATGGCCATCAATCTCTTGAATTGATGGCCATTCATGATGGTCG sense-1 GATCCGCTCCTTGGTTACAATCCTCAATTCAAGAGATTGAGGATTGTAACCAAGGAGTTTTTTGC anti-sense-1 GGCCGCAAAAAACTCCTTGGTTACAATCCTCAATCTCTTGAATTGAGGATTGTAACCAAGGAGCG sense-2 GATCCGTCCGGTCAGTTCTATGACCAATTCAAGAGATTGGTCATAGAACTGACCGGATTTTTTGC anti-sense-2 GGCCGCAAAAAATCCGGTCAGTTCTATGACCAATCTCTTGAATTGGTCATAGAACTGACCGGACG |
| Table S2. qRT-PCR primer used in mouse experiments | | | |
| --- | --- | --- | --- |
| Target genes | Forward primer 5’-3’ | Reverse primer 5’-3’ | Product size (bp) |
| PL-1 Tpbpa PECAM-1 SynA SynB Ctsq Gcm-1 β-actin | GAGGCAGTCCACGAAAC CACAGCCAGTTGTTGAT GTCATGGCCATGGTCGAGTA AGCGTGACAGGCATACTTC GCAGCTGACACCCTCATTAAA AACTAAAGGCCCCATTGCTAC GCTCGGACGACAGGAAC CACGATGGAGGGGCCGGACTCATC | ACCAAGCAGGGTAGTCA CGTTGCCTACCTTGATAC AGCAGGACAGGTCCAACAAC AGGATCGTCTGGGTGGAG TGAGCCTTAGGAGGCTGAGAT CAATCCCCATCGTCTACCC GCGGGTCTCAGGTAAATC TAAAGACCTCTATGCCAACACAGT | 250 112 169 119 218 202 160 252 |
